# Trabecular metal versus non-trabecular metal acetabular components for acetabular revision surgery

**DOI:** 10.1097/JS9.0000000000000615

**Published:** 2023-07-18

**Authors:** Jiajia Lu, Nan Lu, Qiang Fu, Xiaojian Shi

**Affiliations:** a Department of Orthopedic Trauma Surgery, Shanghai Fourth People’s Hospital; b Department of Orthopedic Trauma Surgery, Shanghai Changzheng Hospital, Shanghai; c Department of Orthopedic Trauma, Haimen People’s Hospital of Jiangsu Province, Nantong, Jiangsu, People’s Republic of China

*Dear Editor*,

We are interested in an article entitled ‘Trabecular metal versus non-trabecular metal acetabular components for acetabular revision surgery: A systematic review and meta-analysis’ in your prestigious journal^1^. This meta-analysis included 13 864 total hip arthroplasty revisions in patients who underwent acetabular revision surgery with trabecular metal (TM) (*n*=5619) or non-TM cups (*n*=8245) to compare the efficacy and adverse events of both different acetabular components in acetabular revision surgery. While conducting the meta-analysis, several important questions were addressed and considered.

First, in the study results, it was found that there was no significant difference in cup survival using re-revision for any reason or aseptic loosening as the endpoint following acetabular revision surgery with TM or non-TM cups. The incidence of aseptic loosening and infection was significantly lower, but the incidence of dislocation was significantly higher for TM compared to non-TM cups. Even if the included studies were all non-randomized controlled trials, there was a large sample size in these studies to be analyzed. It identified the advantage and disadvantages of the usage of TM and non-TM in acetabular revision surgery, which provided the reference data for orthopedic surgeons to select which acetabular components. Nevertheless, it should conduct a trial sequential analysis to evaluate whether the sample size is enough to get the true result^2^.

Second, in the section on statistical analysis, the authors conducted a random-effect model on *I* ^2^ value greater than 50% that is judged as the high heterogeneity between studies. However, Chapter 10 in the Cochrane Handbook indicated that the application of a random-effect or fixed-effect model should not be based on an *I* ^2^ value greater than 50% but the heterogeneity between studies on the difference of clinical data in each study from the reviewers’ initial judgment^3^.

Third, we did not see there were subgroup analysis results on the different study designs as a retrospective cohort study, retrospective comparative study, comparative study, and propensity score-matched study. In addition, a subgroup analysis should be conducted on the difference in sample size and follow-up among studies.

Finally, sensitivity analysis is also missing in this meta-analysis; the current meta-analysis results may be affected by the study with large weight due to the large sample size^4^.

## Ethical approval

Our submitted manuscript does not involve any patients without the ethical approval document.

## Consent

Our submitted manuscript does not involve any patients without the written informed consent documents.

## Sources of funding

There was no funding support for this research.

## Author contribution

J.L., N.L., and Q.F.: equally participated in the original idea and writing the first draft; X.S.: edited the final manuscript. All authors approved the last version of this manuscript.

## Conflicts of interest disclosure

The authors declare that they have no conflicts of interest.

## Research registration unique identifying number (UIN)

None.

## Guarantor

All authors.

## Data availability statement

Data sharing is not applicable to this article as no new data were created or analyzed in this study.

## Provenance and peer review

Commentary, internally reviewed.
